# Detailed Study of the Influence of InGaAs Matrix on the Strain Reduction in the InAs Dot-In-Well Structure

**DOI:** 10.1186/s11671-016-1339-3

**Published:** 2016-03-01

**Authors:** Peng Wang, Qimiao Chen, Xiaoyan Wu, Chunfang Cao, Shumin Wang, Qian Gong

**Affiliations:** State Key Laboratory of Functional Materials for Informatics, Shanghai Institute of Microsystem and Information Technology, Chinese Academy of Sciences, Shanghai, 200050 China; Department of Microtechnology and Nanoscience, Chalmers University of Technology, 41296 Gothenburg, Sweden; School of Physics, University of Chinese Academy of Sciences, 19 Yuquan Road, Beijing, 100049 China

**Keywords:** Quantum dots, InAs/InGaAs, Dot-in-well, InGaAs matrix, Photoluminescence, Finite element, AFM, TEM

## Abstract

InAs/InGaAs dot-in-well (DWELL) structures have been investigated with the systematically varied InGaAs thickness. Both the strained buffer layer (SBL) below the dot layer and the strain-reducing layer (SRL) above the dot layer were found to be responsible for the redshift in photoluminescence (PL) emission of the InAs/InGaAs DWELL structure. A linear followed by a saturation behavior of the emission redshift was observed as a function of the SBL and SRL thickness, respectively. The PL intensity is greatly enhanced by applying both of the SRL and SBL. Finite element analysis simulation and transmission electron microscopy (TEM) measurement were carried out to analyze the strain distribution in the InAs QD and the InGaAs SBL. The results clearly indicate the strain reduction in the QD induced by the SBL, which are likely the main cause for the emission redshift.

## Background

Self-assembled quantum dots (QDs) utilized as an active region has provided a unique and flexible approach for fabricating hetero-epitaxy photoelectric devices, offering many advantages compared to the quantum well (QW) structure [1]. InAs dot-in-well (DWELL) lasers on GaAs substrates reveal particular importance due to its great potential in the application of optical fiber communication requiring light wavelength range of 1.3–1.6 μm [2]. Integration of InAs DWELL structure and silicon substrate has been realized by using germanium (Ge, only 0.08 % mismatch with GaAs) as an intermediate layer, which is very significant for the achievement of Si-based photoelectric integration [3, 4]. InAs/InGaAs DWELL superluminescent light-emitting diode and room temperature (RT) continuous-wave (CW)-operated lasers have been fabricated on Si and Ge-on-Si substrates, respectively [5–7]. As described by its name, the DWELL structure consists of a InAs QD layer embedded in the InGaAs matrix, i.e., the InGaAs QW. By the dot layer, the InGaAs matrix is split into two parts. The part above the dot layer in the growth direction is usually called the strain-reducing layer (SRL), while the part below the dot layer is called the strained buffer layer (SBL). The InGaAs SRL is identified to be responsible for the redshift of the InAs QD optical emission as its strain-reducing effect to the InAs QD. Studies of the dependence of optical properties on the InGaAs SRL have been carried out previously, e.g., influence of In composition in InGaAs SRL on the structural and optical properties of InAs QDs has been studied by H.Y. Liu et al. [8]. Muhammad Usman et al. [9] also made a detailed study of the strain-reducing effect caused by the InGaAs SRL and identified the strain reduction as the dominating factor for the redshift of InAs QD photoluminescence (PL) emission. InAlAs and GaAsSb were also tried as the SRLs for reducing the compressive strain of the InAs QD. Although more effective at wavelength adjustment compared with InGaAs SRL (1.7-μm PL emission has been achieved), applying InAlAs and GaAsSb SRL was found to deteriorate the device performance [10–13]. In contrast, the SBL of the DWELL structure has been seldom studied while its importance could not be neglected. It was found that the SBL facilitates the growth of high-density QDs [14]. L. Seravalli et al. have investigated the effect of relaxed InGaAs layer instead of the InGaAs SBL on tuning of PL emission energy of InAs QDs [15, 16]. The studies of Jin Soo Kim et al. and O. Nasi et al. have involved the entire InGaAs matrix including the SRL and SBL experimentally; nevertheless, just a few discrete data were analyzed [17, 18] for the SBL. Specific detailed research on emission energy tuning effect of InGaAs matrix, especially the InGaAs SBL, still need to be carried out.

In this work, the InAs DWELL structure has been studied with the systematically varied InGaAs thickness, especially the SBL thickness. The dependence of the PL properties of the DWELL structure on the thickness of SBL and SRL was investigated in detail. Finite element analysis (FEA) simulation and transmission electron microscopy (TEM) measurement indicate that the reduction of the compressive strain in the InAs QD is enhanced with the increase in the thickness of the SBL layer. It is found that applying both of the InGaAs SBL and SRL may reduce the compressive strain in the InAs QD, leading to a remarkable redshift in the PL emission.

## Methods

InAs/InGaAs DWELL samples were grown on GaAs (100) substrate by VG V90 gas source molecular beam epitaxy (GS-MBE). The grown structure was started with a 100-nm GaAs buffer layer grown at 580 °C after native oxide desorption. Then, the substrate temperature was lowered to 500 °C to grow the DWELL structure and a 3-nm GaAs cap layer. After that, the substrate temperature was raised up to 580 °C for an annealing process of 10 min, followed by growing a 100-nm GaAs cap. The DWELL structure consists of an In_0.12_Ga_0.88_As SBL, a QD layer formed by 2.2 monolayers (MLs) InAs, and an In_0.12_Ga_0.88_As SRL. For morphology analysis of the QD layer, an In_0.12_Ga_0.88_As SBL and 2.2 MLs InAs was repeated at 500 °C on the surface. The thickness of InGaAs SBL and SRL was systematically varied. PL spectra were measured with a Nicolet Magna 860 Fourier transform infrared (FTIR) spectrometer from Thermo Fisher Scientific Inc, equipped with a liquid-nitrogen-cooled InSb detector and a CaF_2_ beam splitter. Samples were excited by a diode-pumped solid state laser (*λ* = 532 nm), and the double modulation mode was used to eliminate the mid-infrared background radiation over 2 μm. The InAs QD morphology was measured by a Bruker Icon atomic force microscopy (AFM) in the tapping mode. The scanned area was 2 × 2 μm^2^. We have also measured the TEM micrograph of InAs QD assembled on the InGaAs SBL.

In order to analyze the strain reduction in the InAs QD induced by the InGaAs SBL, finite element simulation was carried out. The model consists of InAs/InGaAs DWELL structure on a GaAs buffer layer with one InAs QD included. The InAs QD is built in a spherical crown shape with a diameter of 40 nm and height of 5 nm. The InAs QD is located at the center of the 100 × 100 nm^2^ square DWELL structure, which corresponds to the InAs QD density measured by AFM. In the simulation, lattice constant of the border is fixed, and a relaxation process is conducted on the InAs/InGaAs DWELL region. The strain distribution in the QD and the SBL was revealed by the simulation.

## Results and Discussion

**Fig. 1:**
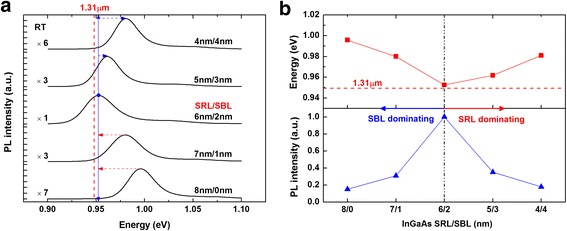
Room temperature PL spectra (**a**) and its intensity, energy information (**b**) of InAs QDs embedded in total thickness fixed InGaAs matrix

Firstly, we investigated the first group of samples with a total InGaAs layer thickness fixed at 8 nm, while the InAs QD layer was inserted in the InGaAs matrix at a different depth in the growth direction. Five InAs/InGaAs DWELL samples were prepared with the SBL thickness varying from 0 to 4 nm in step of 1 nm, while the corresponding SRL thickness varying from 8 to 4 nm. RT PL spectra of the DWELL structure were shown in Fig. 1a. Strong emissions at infrared band were observed for all samples. Ground state (GS)-related emissions are verified to dominate all the spectra. A redshift followed by a blueshift behavior of the PL emission was observed when the InAs QD layer was moved from the bottom of the InGaAs matrix upwards in the growth direction. The red line marked the interesting wavelength of 1.31 μm. The peak energy and intensity of the PL spectra are summarized in Fig. 1b, and two distinct regions are classified and marked by red and blue arrows. Previous study has revealed that thickness increase of the InGaAs SRL leads to redshift of the PL peak and intensity enhancement, which is corresponding to SRL-dominating region marked by the red arrow at the right side [9]. However, a reversed tendency is observed for the region at the left side, which is named as the SBL-dominating region. A sharp redshift of the PL peak was observed when the SBL thickness was increased from 0 to 2 nm, and a strong enhancement in PL intensity was also observed. Therefore, the SBL layer plays an important role similar to the SRL in the wavelength tuning of the InAs QDs. In addition, we also carried out the low-temperature (LT) PL measurement and the developing behavior of the PL intensity and linewidth; energy is consistent with that at RT.

**Fig. 2:**
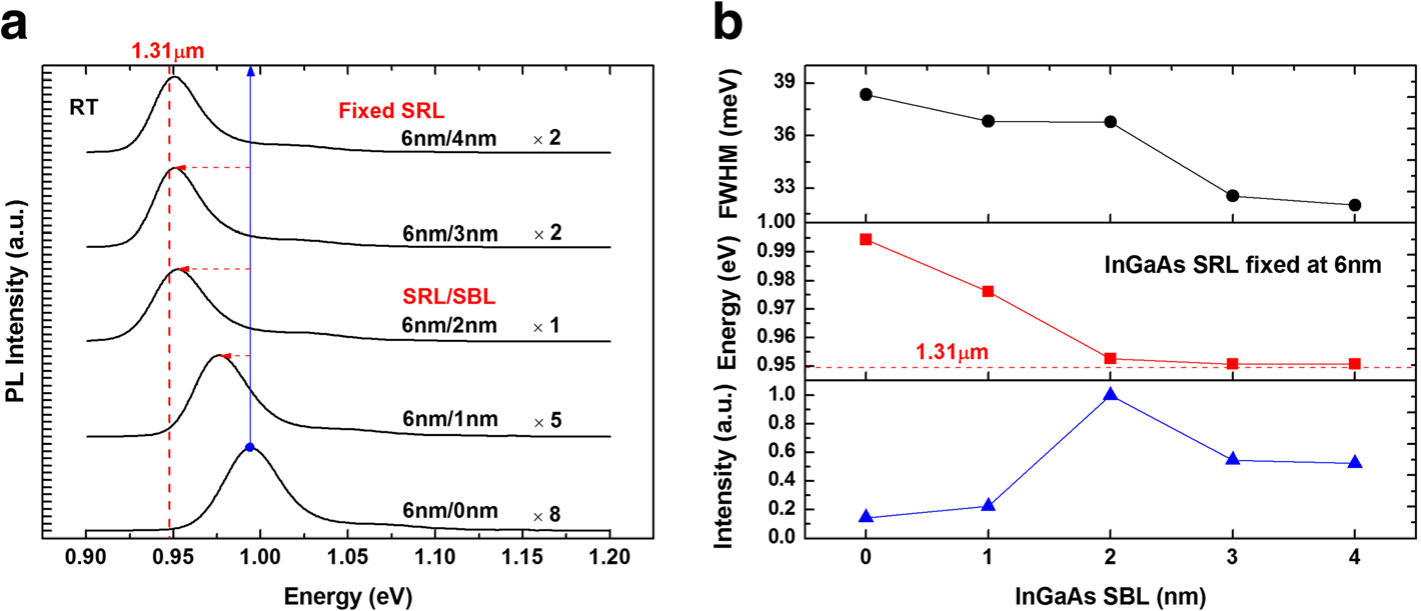
Room temperature PL spectra (**a**) and its intensity, energy, and FWHM information (**b**) of InAs QDs embedded in SRL fixed InGaAs matrix

The above results suggest it is very necessary to study the influence of SBL on the PL properties of the DWELL structure. Five InAs/InGaAs DWELL samples were investigated with the SRL thickness fixed at 6 nm, while the SBL thickness was varied from 0 to 4 nm. The PL spectra were shown in Fig. 2a. Redshift of PL peaks was indeed observed when the SBL thickness was increased. The dependence of peak wavelength, intensity, and full width at half maximum (FWHM) on the SBL thickness was shown in Fig. 2b. Remarkable redshift of the PL peak was obtained when the SBL thickness was increased from 0 to 2 nm, and a saturation behavior was observed when the SBL thickness was further increased. It is found that a 2-nm InGaAS SBL results in a remarkable 42-meV energy drop in the PL peak energy of the DWELL structure. As shown in Fig. 2b, the peak intensity was enhanced with the SBL thickness in the range of 0–2 nm and started to decrease when the SBL thickness was above 2 nm. It is noteworthy that the PL linewidth of the DWELL structure continuously decreases with the increase in SBL thickness.

**Fig. 3:**
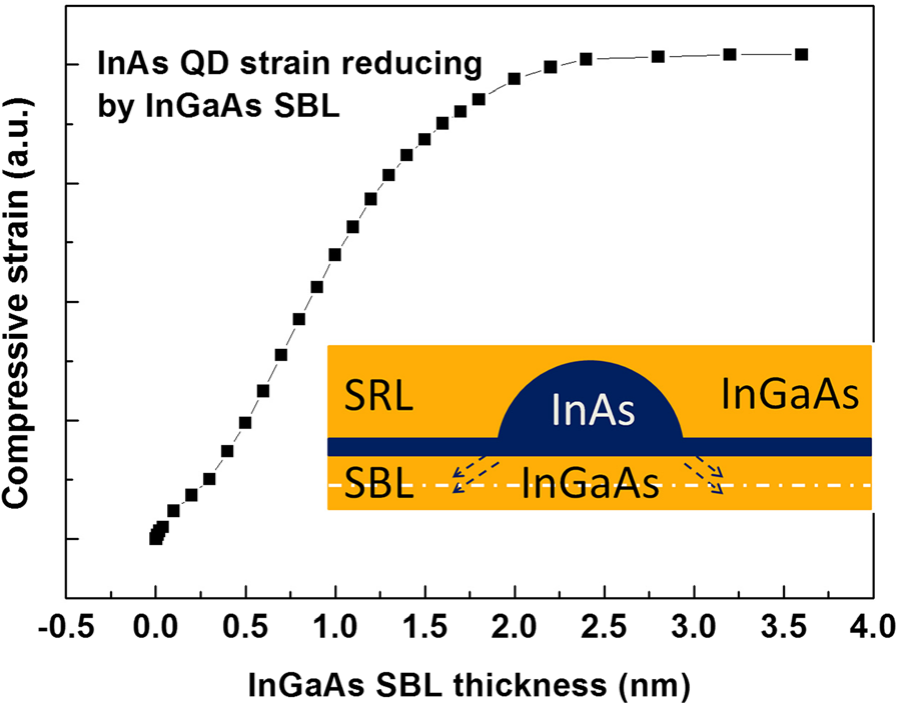
Calculated compression strain of InAs QD as a function of InGaAs SBL thickness and the simulation schematic diagram

Previous study has confirmed that the InGaAs SRL is effective at reducing the internal compressive strain in the InAs QDs, which is the primary cause of PL emission redshift [19]. As mentioned above, the InGaAs SBL also leads to redshift of the PL peak, likely through the same mechanism as that of the SRL. In spite of being fully strained, InGaAs SBL is also expected to reduce the internal compressive strain in the InAs QDs. In order to verify this fact, a simulated calculation was carried out using FEA. The InAs QD is defined in a spherical crown shape (width 40 nm, height 5 nm) and is enveloped by the InGaAs matrix (SBL down and SRL up), as shown in the inset of Fig. 3. The thickness of InGaAs SRL is fixed at 6 nm. The statistical average compressive strain in the InAs QDs is recorded from the simulated result with the increase of the InGaAs SBL thickness. As shown in Fig. 3, the compressive strain in the InAs QDs is observed to be reduced continuously followed by a saturation behavior as the InGaAs SBL thickness up to 2.4 nm, which is consistent with the above experiment result shown in Fig. 2b. Therefore, both of the SBL and SRL in the DWELL structure induce strain reduction in the InAs QD, leading to redshift of the PL emission.

**Fig. 4:**
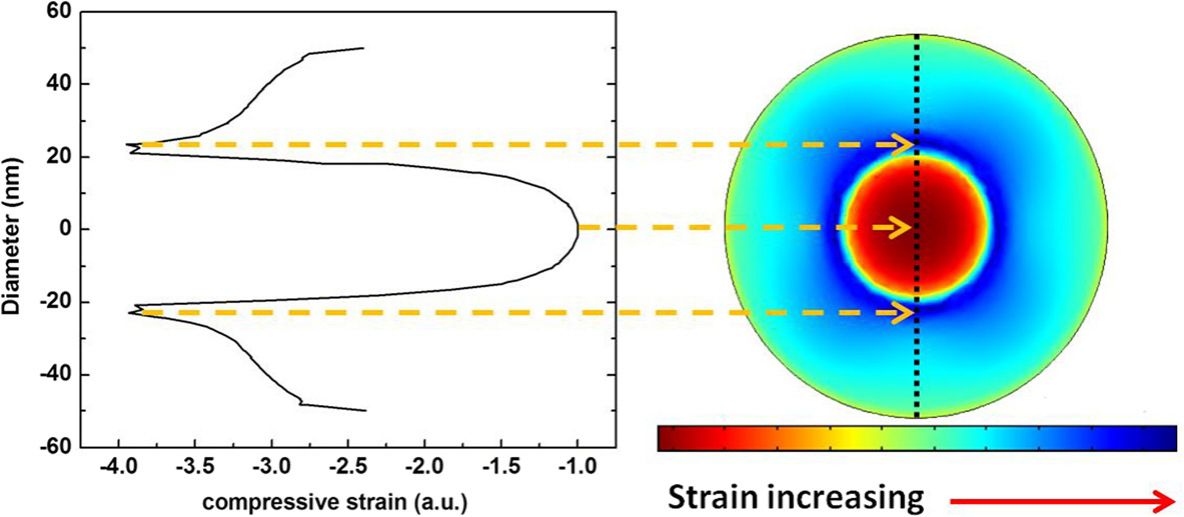
In-plane compression strain distribution map of InGaAs SBL (*right*) and the compression strain distribution on the *dashed line* (*left*)

In order to have a better understanding of the strained system, in-plane strain distribution of InGaAs SBL is shown in Fig. 4 (the cross section position is marked by the dash-dot line in the inset of Fig. 3), and the strain distribution scan on the dashed line is also shown. As can be seen, all the region of InGaAs SBL is still in compressive strain and a specific strain distribution is revealed. The red circular area, which has the minimum compressive strain, is matched with the location of InAs QD. Outward, a deep blue belt is surrounding, which has the maximum compressive strain. Therefore, the strong compressive strained InAs QDs tend to expand (shown by the dashed arrows in the inset of Fig. 3), through the deformation of the SBL exactly underneath. This process is revealed by the redistribution of the compressive strain in the InGaAs SBL shown in Fig. 4.

**Fig. 5:**
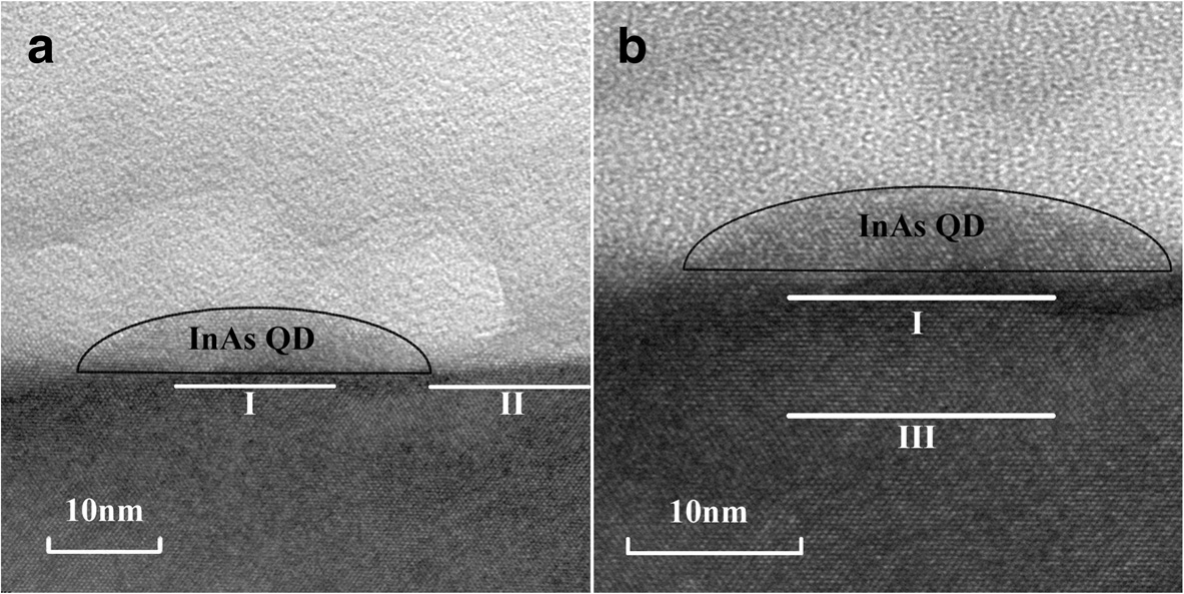
TEM micrograph of InAs QD assembled on a InGaAs strain buffer layer. Lattice constants below and beside the InAs QD are compared indicated by line I and II (**a**), lattice constants below the InAs QD with different depth are compared indicated by line I and III in the enlarged image (**b**)

For a further direct-viewing investigation to the compressive strain-reducing effect, we have measured the TEM micrograph of InAs QD assembled on the InGaAs SBL as shown in Fig. 5. Two white lines in Fig. 5a are located in the InGaAs SBL, and line I is just underneath the InAs QD. The ratio for the lattice constant between line I and line II is 1.04. For Fig. 5b, another line III is added in the GaAs buffer layer far beneath the InAs QD, and the lattice constant ratio between line I and line III is 1.03. A dark band, which indicates the disorder of crystal lattice, is observed under the InAs QD. This should be caused by the abovementioned deformation of the InGaAs SBL. These observations are direct evidences that the lattice of the compressive strained InGaAs SBL has been stretched leading to strain reducing of the overgrown InAs QD, which is consistent with the above simulation result.

**Fig. 6:**
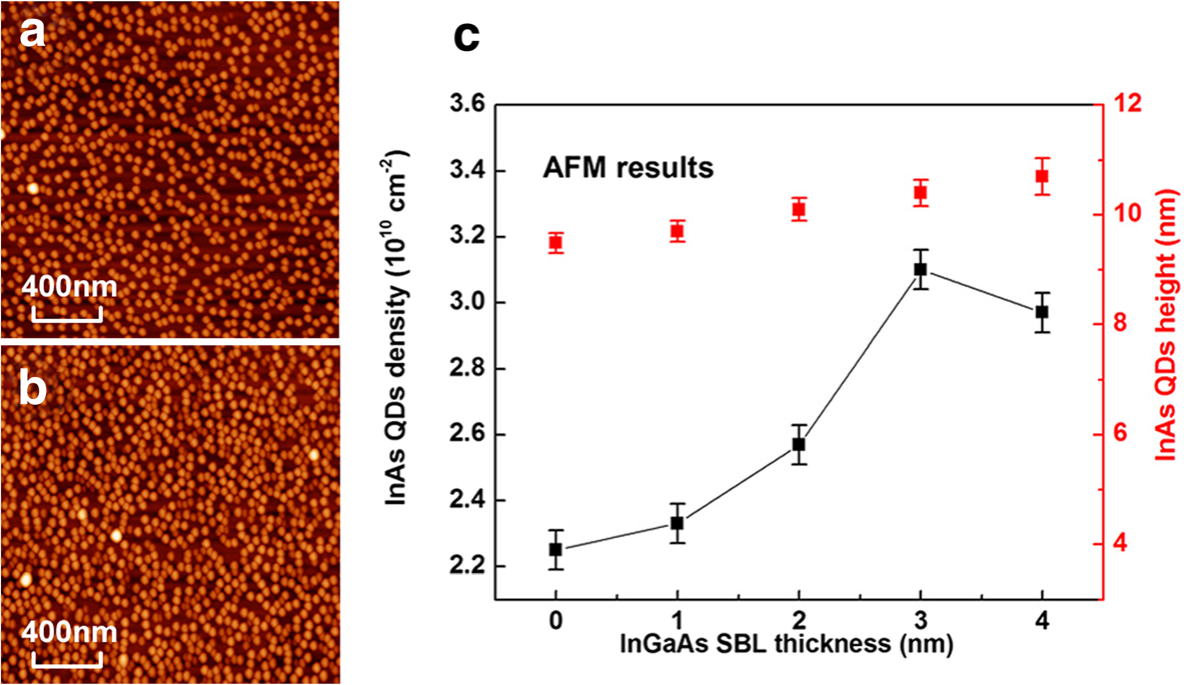
AFM diagrams of InAs QDs overgrown on GaAs (**a**) and InGaAs SBL (**b**) and dependence of QD density and height on InGaAs SBL thickness (**c**)

In addition to the strain reduction effect, the SBL may facilitate the growth of high-density InAs QDs. AFM images shown in Fig. 6a, b indicate that uniform InAs QDs are formed on both GaAs and InGaAs SBL. Density of the InAs QDs grown on InGaAs SBL is obviously larger than that on GaAs. The dependence of the QD density on the SBL thickness is shown in Fig. 6c. It is found that the InAs QD density rises continuously with the InGaAs SBL thickness up to 3 nm.

The redshift behavior of InAs QD PL emission has been investigated as a function of the QD height previously [20]. As shown in Fig. 6b, a 1.0-nm increase in the average QD height is observed as the SBL thickness is changed from 0 to 4 nm. Such an increase in the QD height can only cause a redshift less than 5 meV as the quantum confinement effect becomes weak [20]. Thus, the dominating reason for the redshift of PL emission is the reduction of compressive strain in the QD induced by the SBL and SRL as discussed before.

**Fig. 7:**
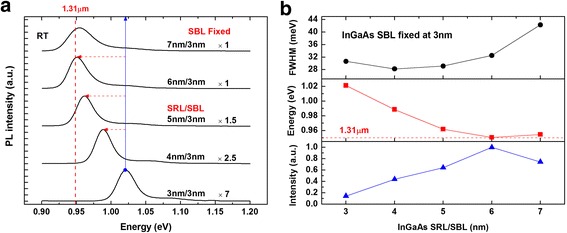
Room temperature PL spectra (**a**) and its intensity, energy, and FWHM information (**b**) of InAs QDs embedded in SBL fixed InGaAs matrix

Finally, the dependence of the emission wavelength on the thickness of SRL is investigated. Six InAs/InGaAs DWELL samples were studied with the SBL thickness fixed at 3 nm, while the SRL thickness is raised from 3 to 7 nm. The PL spectra are shown in Fig. 7a. The PL peak undergoes redshift continuously as the SRL thickness is changed from 3 to 6 nm. Saturation behavior is observed when the SRL thickness is further increased from 6 to 7 nm. The PL results are summarized in Fig. 7b. More than 70-meV redshift is obtained when the SRL thickness is changed from 3 to 6 nm. Moreover, the PL peak intensity rises linearly with the SRL thickness up to 6 nm and then declines slightly for the sample with 7 nm SRL. For the samples with SRL thickness below 6 nm, their PL peak linewidths are distributed closely. However, sudden broadening of the PL emission is observed for the sample with a 7-nm SRL, indicating that the sample quality deteriorates slightly, likely due to the too large compressive strain in the whole DWELL structure. This observation is consistent with the previous study [9] and provides further support for our experiment result.

## Conclusions

InAs/InGaAs DWELL structures with a varied InGaAs matrix were grown by GS-MBE on GaAs (100) substrate. Both of the InGaAs SRL and SBL are observed to be responsible for the redshift of PL emission. The dependence of the redshift on the SBL or SRL thickness obeys a linear function followed by a saturation behavior. It is found that the PL intensity can be greatly enhanced by applying the SRL and SBL. FEA simulation results reveal that the InGaAs SBL can also reduce the compressive strain in the InAs QD, while the same effect was reported previously for the InGaAs SRL, resulting in a remarkable redshift in the PL emission. TEM measurement results also confirmed that InGaAs SBL reduces the compressive strain of InAs QD through lattice deformation. This detailed study of wavelength tuning effect of the InGaAs matrix on the PL emission of the DWELL structure is very meaningful for the InAs QD device applications.

## Abbreviation

AFM: atomic force microscopy
CW: continuous-wave
DWELL: dot-in-well
FEA: finite element analysis
FWHM: full width at half maximum
GS: ground state
GS-MBE: gas source molecular beam epitaxy
PL: photoluminescence
QD: quantum dot
RT: room temperature
SBL: strained buffer layer
SRL: strain-reducing layer
TEM: transmission electron microscopy
